# Design of a hybrid AI network circuit for epilepsy detection with 97.5% accuracy and low cost-latency

**DOI:** 10.3389/fphys.2025.1514883

**Published:** 2025-03-26

**Authors:** Liufang Sheng, Xuanxu Chen, Yuejun Zhang, Ke Yan, Junping Chen, Zhikang Chen, Hanyu Shi, Yi Gong

**Affiliations:** ^1^ The Affiliated People’s Hospital, Ningbo University, Ningbo, China; ^2^ Faculty of Electrical Engineering and Computer Science, Ningbo University, Ningbo, China; ^3^ Department of Anesthesiology, Ningbo No. 2 Hospital, Ningbo, China

**Keywords:** epilepsy detection, biomedical diagnostics, hybrid AI network model, convolutional neural network, hardware implementation

## Abstract

Epilepsy detection using artificial intelligence (AI) networks has gained significant attention. However, existing methods face challenges in accuracy, computational cost, and speed. CNN excel in feature extraction but suffer from high computational latency and power consumption, while SVM rely heavily on feature quality and expensive kernel computations, limiting real-time performance. Additionally, most CNN-SVM hybrid model lack hardware optimization, leading to inefficient implementations with poor accuracy-latency trade-offs. To address these issues, this paper designs a hybrid AI network-based method for epilepsy detection using electroencephalography (EEG) signals. First, a hybrid AI network was constructed using three convolutional layers, three pooling layers, and a Gaussian kernel SVM to achieve EEG epilepsy detection. Then, the design of the multiply-accumulate circuit was completed using a parallel-style row computation method, and a pipelined convolutional computation circuit was used to accelerate the convolutional computation and reduce the computational overhead and delay. Finally, a single-precision floating-point exponential and logarithmic computation circuit was designed to improve the speed and accuracy of data computation. The digital back-end of the hardware circuit was realized under the TSMC 65 nm process. Experimental results show that the circuit occupies an area of 3.20 mm^2^, consumes 4.28 mW of power, operates at a frequency of 10 MHz, and has an epilepsy detection latency of 0.008 s, which represents a 32% reduction in latency compared to those reported in the relevant literature. The database test results showed an epilepsy detection accuracy of 97.5%, a sensitivity of 97.6%, and a specificity of 97.2%.

## 1 Introduction

Epilepsy is a common chronic neurological condition characterized by irregular firing of neurons in the brain, resulting in transient impairment of brain function. This can result in abnormal behavior or even loss of consciousness in affected individuals (Falco-Walter, 2020). Affecting more than 65 million people worldwide, with a lifetime prevalence of 3%, epilepsy can occur across all age groups, but is most commonly seen in infants and individuals over the age of 50 (Balestrini et al., 2021; Sen et al., 2020). While approximately 70% of patients can control their seizures with appropriate treatment, the remaining 30% suffer from drug-resistant epilepsy. For these individuals, seizures are unpredictable and can be life-threatening, especially when they occur in hazardous situations (Milligan, 2021; Royer et al., 2022).

**FIGURE 1:**
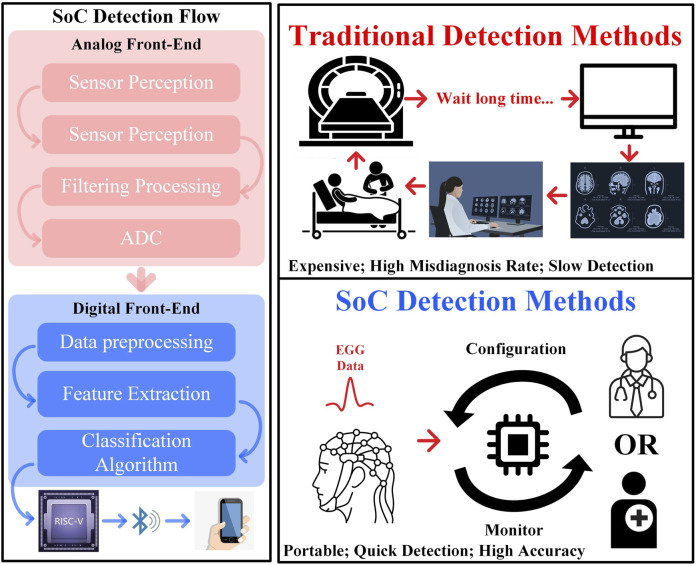
The epilepsy detection process.

Therefore, detection of epilepsy is of utmost importance and can be categorized into traditional detection methods and Systemon-Chip (SoC)-based detection methods as illustrated in Figure 1. Traditional detection methods require the patient to undergo testing with the aid of a specialized doctor and medical devices. This typically involves MRI or EEG scans, followed by a waiting period of several days to obtain the results. However, since epileptic patients do not usually exhibit abnormal discharges under normal conditions, hospital-based tests can be slow and may fail to accurately diagnose the condition. This makes the real-time nature of SoC-based diagnostic methods particularly valuable. The SoC diagnostic approach consists of three main components. The first is the analog front-end, where a sensor amplifies the signal through a filter and converts it to an EEG *via* an Analog-to-Digital Converter (ADC). Then, the digital section processes the data through pre-processing, feature extraction, and classification algorithms. Finally, the diagnostic results are transmitted to a mobile device and the outcome of the epileptic seizure is uploaded to the nearest hospital for further analysis.

In epilepsy detection research, there are primarily two categories of software algorithms: those that rely on the use of machine learning algorithms to classify manually constructed EEG features, and those that utilize artificial neural networks to classify manually or neural network-constructed features. In terms of machine learning, Ru et al. (2022) proposed a method for detecting epilepsy in noisy environments by extracting the variational mode decomposition eigenmode function and the improved sample entropy as input features, which are classified using a random forest. Fredes et al. (2024) proposed an approach that integrates the discrete wavelet transform with an SVM model. This method consists of first filtering the raw EEG signals, followed by isolating particular frequency bands and employing the discrete wavelet transform to derive their characteristics, and culminating in classification *via* the SVM model. In the realm of artificial neural networks, Tawhid et al. (2022) proposed a deep learning framework grounded in Convolutional Long Short-Term Memory (Conv-LSTM) for identifying epilepsy from EEG signals. This framework has surpassed the existing top-tier outcomes on the dataset in question, demonstrating its efficacy as an automated diagnostic system for epilepsy. Mathew et al. (2023) introduced a feature integration technique that leverages Variational Mode Decomposition (VMD) to discern different types of epileptic seizures from scalp electroencephalogram (EEG) readings. Additional related studies based on machine learning and artificial neural networks are summarized in Table 1.

**TABLE 1:** Related works about epilepsy detection.

Study	Feature	Classifiers	Performance		
			Acc	Sen	Spe
Ru et al. (2024a)	N/A	1D-CNN	96.06%	95.48%	N/A
Ru et al. (2024b)	STFT	2D-CNN	92.89%	96.17%	94.41%
Ullah et al. (2018)	N/A	1D-CNN	99.10%	N/A	N/A
Malekzadeh et al. (2021)	Statistical Feature	CNN-RNN	99.13%	98.96%	98.96%
Wu et al. (2024)	N/A	DTGCN	98.00%	N/A	N/A
Song et al. (2024)	STFT	LSTM	96.59%	N/A	N/A
Tang et al. (2024)	Path Signature	Bi-LSTM	94.84%	91.05%	98.63%
Najafi et al. (2022)	LB&DWT	RNN-LSTM	96.10%	96.80%	97.40%
Shen et al. (2024)	STFT	Google-net CNN	97.74%	98.90%	N/A

Abbreviations: Acc, accuracy; Sen, sensitivity; Spe, specificity; N/A, not analyzed; STFT, short-time fourier transform; RNN, recurrent neural network; LB, longitudinal bipolar montage; DWT, discrete wavelet transform; CNN, convolutional neural networks; DTGCN, dynamic temporal graph convolutional network; Bi-LSTM, bidirectional long short-term memory.

In terms of hardware implementation, Zhang et al. (2021) developed a tailored closed-loop system for epilepsy management, featuring initial learning coupled with real-time adjustments. However, the model lacked generalization capability. Huang et al. (2020) developed a low-power SVM processor with on-chip active learning for real-time epileptic seizure control, leveraging parallel computing and hardware-shared CORDIC-based processing. However, its detection latency of 0.71 s is relatively high compared to FPGA-based implementations. Pelkonen et al. (2020) developed a modular brain chip, called the Modular Platform for Epilepsy Modelling *in Vitro* (MEMO), which allows researchers to simulate localized epileptic seizures a feat that has been difficult to achieve in previous studies. Liu et al. (2024) proposed a SoC for seizure monitoring that includes a lossless neural recording system with a dynamic range up to 84.9 dB and tolerance for stimulation artifacts (SA). Lastly, Cao et al. (2022) introduced a three-dimensional deep network based on a dual-stream attentional mechanism (TSA3-D), primarily for classifying epileptic syndromes in children. This technique leverages multi-channel time-frequency and frequency-space features optimized by multiple montage transformations to reduce artifacts and enhance EEG feature learning.

Although many studies have made significant progress in epilepsy detection, many existing models perform poorly on different datasets and suffer from weak generalization capabilities. This paper aims to design a high-accuracy and low cost-latency hybrid AI network epilepsy detection hardware circuit that enhances the detection ability. The layout of this paper is delineated as follows: The first section provides an overview of the paper. The second section describes our suggested approach in detail. The third section showcases the experimental outcomes and their discussions thereof. The final section wraps up the paper’s conclusion.

The main contents of this paper are summarized as follows: first, a hybrid AI model epilepsy detection hardware circuit was designed to detect epilepsy via EEG signals. A FFT feature extractor was designed to improve accuracy and enhance robustness. To address the computational resource requirements for two-dimensional convolution with three different kernel sizes, a configurable hardware convolution layer was designed to achieve a high degree of hard-ware resource reuse and reduce overhead. Then, pipeline convolution algorithm was used to accelerate the convolution operation, facilitating the construction of the convolution kernel and the final extraction of local features. Finally, a Gaussian-like kernel support vector machine was used to classify epilepsy. The CORDIC computing circuit was designed to accelerate exponential and logarithmic calculations and improve the calculation accuracy, thus realizing the design of the whole epilepsy detection circuit.

## 2 Hybrid AI network epilepsy detection model

### 2.1 Epilepsy EEG dataset

This study utilized two data models: the epileptic EEG dataset from the University of Bonn and the CHB-MIT epileptic EEG dataset available on the PhysioNet portal. The BONN EEG dataset includes data from five epileptic patients and five healthy individuals. It is divided into five subsets as shown in Table 2. Each subset of this single-channel dataset contains 100 data segments, each lasting 24 s, with a sampling rate of 174 Hz (Andrzejak et al., 2001). Among these subsets, datasets *Z* and *O* were collected from five healthy participants with closed and open eyes, respectively, and served as a control group. The other three subsets *N*, *F*, and *S* were collected from epileptic patients. Specifically, dataset *N* was recorded from the hippocampus during the interictal period, dataset *F* was recorded from the epileptogenic zone during the interictal period, and dataset *S* was recorded from the epileptogenic zone during epileptic seizures.

**TABLE 2:** University of Bonn EEG epilepsy dataset Intracranial EEG (Hippocampus).

Dataset	Sample Number	Volunteer State	Data Type Electrode Position
Z	100	Healthy individual	Scalp EEG
O	100	Healthy individual	Scalp EEG
N	100	Epilepsy patient	Intracranial EEG (Hippocampus)
F	100	Epilepsy patient	Intracranial EEG (Focus area)
S	100	Epilepsy patient	Intracranial EEG (Focus area)

The CHB-MIT epilepsy EEG dataset is part of an MIT database comprising scalp EEG data from children and adolescents with refractory epilepsy. This dataset includes EEG signals from 23 patients: 17 females between the ages of 2 and 22 years and five males between the ages of 3 and 16 years (Goldberger et al., 2000). A total of 967.85 h of scalp EEG data were recorded from the CHB-MIT dataset, with data collection times ranging from 1 to 4 h per patient. The objective was to detect individual epileptic seizures by classifying the data into interictal (between seizures) and ictal (during seizures) periods. For each data sample in the CHB-MIT dataset, the samples were initially categorized into interictal and ictal groups. Each data sample, spanning several hours, was segmented into 20-s intervals to form the EEG input data.

### 2.2 Hybrid AI network model

**FIGURE 2:**
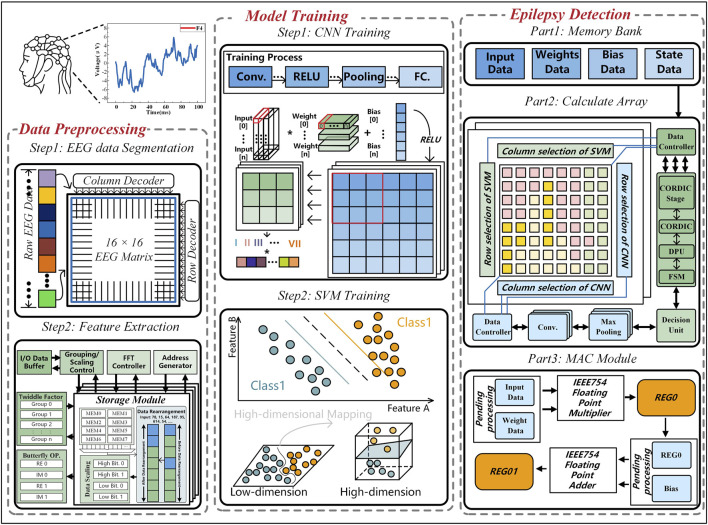
Hybrid AI network for epilepsy detection.

The hybrid AI network model is shown in Figure 2. The main process of the hybrid AI neural network circuit is divided into three stages: data preprocessing, model training, and epilepsy detection hardware circuit design. In the data preprocessing stage, the EEG dataset is segmented into 256-point frames, ensuring that both seizure and non-seizure events are sufficiently represented. These segmented EEG frames are then fed into a feature extraction module. FFT is applied to transform the time-domain signals into frequency-domain signals. From the FFT spectrum, we extract the amplitude spectrum, which provides information on the magnitude distribution of different frequency components. This feature is crucial for capturing epileptic signal characteristics, as seizures often exhibit distinct frequency patterns.

The FFT module consists of an FFT controller, an address generator, a grouping controller, an I/O buffer, a twiddle factor, a butterfly unit, and a storage module. During epileptic seizures, the EEG signals of patients exhibit increased high-frequency components and decreased low-frequency components compared to normal states. Typical epileptiform discharges, such as spikes and sharp waves, also emerge. By using FFT as a feature extractor, the voltage of the EEG signal is transformed from the time domain to the frequency domain, and the frequency domain spectrum is extracted to further improve the detection accuracy and improve the robustness of the epilepsy detection system. Within the storage module, discontinuous data are reorganized into continuous data, and both high and low-dimensional data are processed separately to enhance the readability of epileptic signals.

In the model training stage, the trained model is a CNN-SVM hybrid AI model, which includes three convolutional layers, three pooling layers, and ReLU activation functions. The final fully connected layer is replaced by an SVM classifier, a supervised learning algorithm that finds an optimal hyperplane in the feature space to improve generalization performance. To enhance the accuracy of epilepsy classification, the SVM employs a quasi-Gaussian kernel function for feature mapping.

Finally, in the epilepsy detection hardware circuit design, the system is divided into a memory module and a computation module. The memory module stores input data, weight data, bias data, and state data, while the computation module consists of CNN and SVM components, with processing elements (PE) shared between CNN and SVM to optimize circuit area and improve computational efficiency.

The selection of the Gaussian kernel SVM and the three-layer CNN configuration in this work is grounded in well-established theoretical principles. The Gaussian kernel SVM is chosen for its ability to map nonlinear EEG features into a higher-dimensional space, where seizure and non-seizure patterns become more separable. This approach aligns with Vapnik-Chervonenkis (VC) theory, ensuring optimal generalization while mitigating overfitting. Meanwhile, the three-layer CNN architecture with 5 × 5, 3 × 3, and 1 × 1 kernels follows a hierarchical feature extraction strategy, capturing both global and localized seizure patterns while maintaining computational efficiency. The progressive reduction in kernel size balances accuracy and hardware complexity, ensuring real-time feasibility. These choices are not arbitrary but are based on deep learning theory, statistical learning principles, and practical considerations for efficient hardware implementation, making the proposed model well-suited for real-time epilepsy detection in resource-constrained environments.

## 3 EEG epilepsy detection circuit design

**FIGURE 3:**
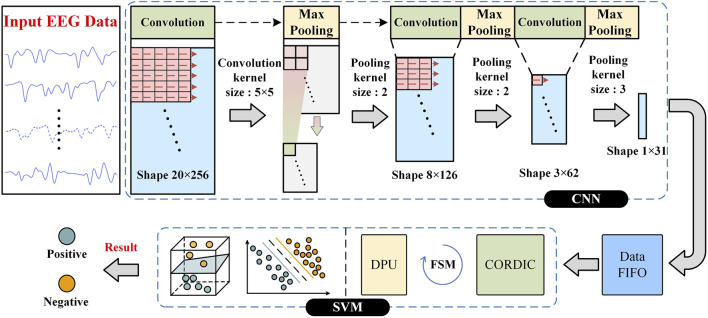
Structure of CNN and SVM epilepsy detection model.

The hierarchical structure of the CNN-SVM-based epilepsy detection model is illustrated in Figure 3. The overall model consists of a CNN cascaded to an SVM. In this setup, the CNN is responsible for feature extraction while the SVM handles epilepsy classification. The input to the CNN is preprocessed EEG signals that have undergone noise removal and other preparatory steps. The data is then passed through convolution layers with kernel sizes of 5 × 5, 3 × 3, and 1 × 1, respectively, with each convolution operation followed by max pooling. The output of the CNN is a 1 × 31 feature vector, which is subsequently fed into the SVM for binary classification to determine whether the input data indicates epilepsy.

In the CNN module, two-dimensional convolution is performed by sliding the convolutional kernel across the entire dataset with a stride of 1. Each weight in the convolutional kernel is multiplied by the corresponding data it has slid over, and the results are summed up to extract relevant features. Max pooling is applied by sliding a pooling window over the dataset with a stride equal to the window size, retaining only the maximum value within the window and discarding the rest, thus achieving feature compression. The SVM component then classifies the data by maximizing the margin between epileptic and non-epileptic features using an optimal hyperplane to achieve accurate classification. The following is the convolutional pooling and SVM circuit structure.

**FIGURE 4:**
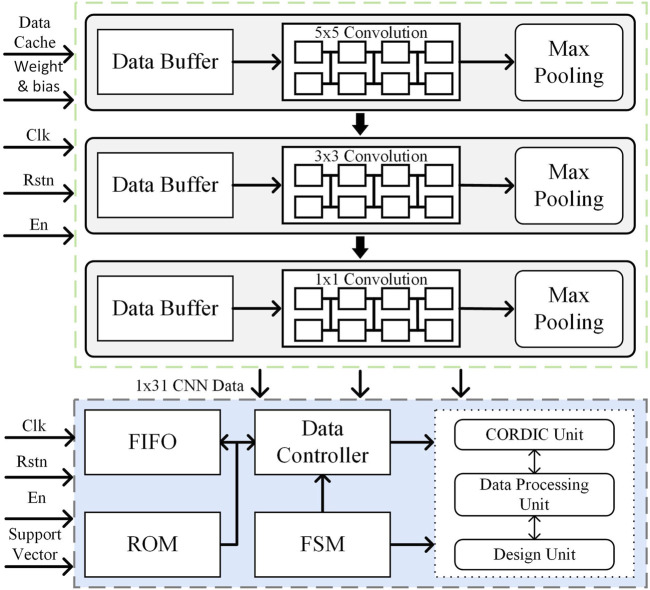
Data flow diagram of hybrid AI network model.

In the proposed hybrid AI network for epilepsy detection, the data flow and interaction structure are depicted in Figure 4. The input consists of EEG signals with a size of 20 × 256, which first enter the CNN module. The EEG data are processed sequentially through three convolutional layers with kernel sizes of 5 × 5, 3 × 3, and 1 × 1, each followed by a max pooling layer, to extract both spatial and temporal features. After each convolution operation, the intermediate feature maps are stored in the Data Buffer. After each convolution, the data is caught in the Data Buffer. When the convolution of this layer ends, the Data Buffer begins to convolve the input data to the next layer. After passing through the CNN, the final output is a 1 × 31 feature vector, representing a high-dimensional feature encoding of the EEG signal. This feature vector is then transmitted to the SVM classifier, which consists of FIFO, ROM, a finite state machine (FSM), a data control unit, a data processing unit, and a CORDIC unit. The feature data are first stored in FIFO for buffering, while the required support vectors and parameters for SVM classification are fetched from ROM. The data control unit ensures seamless synchronization between the CNN feature extraction and the SVM classification process. The Gaussian kernel computation in the SVM involves non-linear operations, such as exponential and logarithmic calculations, which are efficiently handled by the CORDIC computation unit. This optimization enhances the computational efficiency of the Gaussian kernel SVM, making it suitable for real-time EEG classification. Finally, the SVM outputs the epilepsy detection result, determining whether the given EEG data corresponds to a seizure event, thereby completing a single epilepsy detection operation.

The subsequent sections of Chapter three are structured as follows. Section 3.1 describes the configurable, pipelined convolutional hardware circuits proposed in this paper. Section 3.2 describes the principles and circuit design of the maximum pooling computation. Section 3.3 describes the principles of the SVM circuits and the design of the hardware circuits for the hybrid AI network model.

### 3.1 Pipelined convolution circuit

The convolution operation is the most computationally intensive part of a convolutional neural network, directly influencing the detection response speed. In this work, we implement three configurable convolutional layers with kernel sizes of 5 × 5, 3 × 3, and 1 × 1, enabling multi-scale feature extraction while optimizing hardware efficiency. To reduce computational overhead and resource consumption, a configurable hardware architecture is adopted, allowing convolutional layers to dynamically adjust kernel sizes through mode selection, thereby maximizing hardware reuse.

**FIGURE 5:**
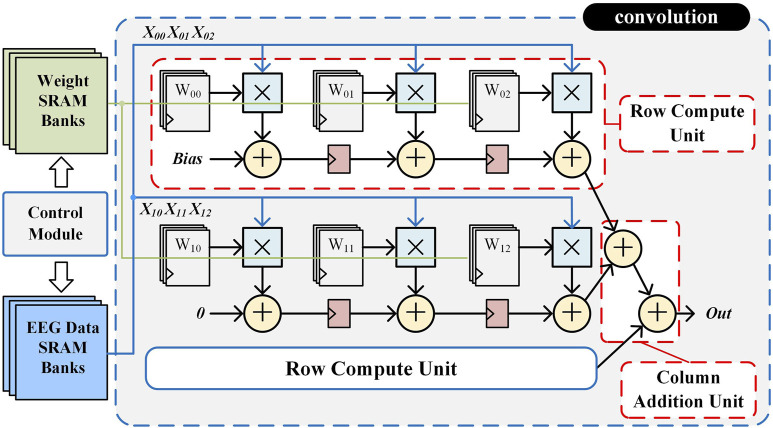
Hardware structure of the 3 × 3 convolution circuit.

To enhance computational throughput, a pipelined convolutional computation circuit is designed to accelerate processing. Taking the 3 × 3 convolutional computing unit as an example, the hardware structure is shown in Figure 5. The design consists of three parallel row operation units, each containing three PE. Each PE is composed of a multiplier, an adder, and a register, forming an efficient multiply-accumulate (MAC) pipeline.

In the initialization stage, the control module reads the weight data from SRAM to the corresponding registers to participate in the convolution operation. In the operation stage, the EEG data are sequentially read out from the SRAM by the control module to participate in the operation. The first data X_00_ is multiplied with W_00_ in the first operation unit and then added with Bias, and passed to the second operation unit through a flip-flop after one clock cycle. Meanwhile, X_01_ is multiplied by W_01_ in the second operation unit and added to the result of the first operation unit from the flip-flop. Similarly, in the third clock cycle, the third operation unit completes the operation and outputs the first row of results X_00_ × W_00_ + X_01_ × W_01_ + X_02_ × W_02_. All three rows are parallelized at the same time and the results are summed to output a single convolutional result. This pipelined accumulation process continues until the full convolution operation is completed, ensuring high-speed execution with minimal latency. By fully parallelizing row computations and summing partial results efficiently, this hardware-optimized convolutional unit significantly reduces computation latency and improves resource utilization. The configurable architecture, combined with pipeline optimization, enables low-power, high-throughput EEG feature extraction, making it particularly suitable for real-time epilepsy detection in resource-constrained environments.

### 3.2 Max pooling circuit

**FIGURE 6:**
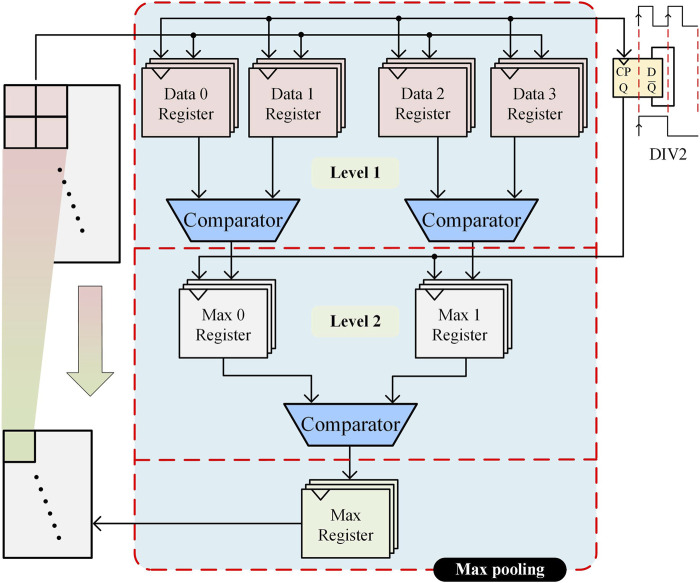
Hardware structure of the pooling circuit.

After the convolution calculation is completed, the system state enters the pooling calculation module, and the hardware circuit architecture of pooling calculation is shown in Figure 6. The pooling module is an important part of the CNN, mainly responsible for the dimensionality reduction and compression of CNN data, reducing the complexity of the corresponding model and reducing the risk of model overfitting. Maximum pooling is adopted in this work, and the pooling module is designed using a two-stage comparison scheme. The first-level comparison module contains four sets of registers and two maximal comparators for pre-comparing two larger values. The second-level comparison module contains two sets of registers and a maximum comparator for comparing the maximum of the two larger values. The pooling calculation is implemented as follows: First, the four numbers in the pooling window are put into the first-level comparison registers according to the base clock frequency. The comparison of Data0 with Data1 and Data2 with Data3 is performed. Then, the two maximum values after the first-level comparison are put into the second-level comparison registers. The second-level comparison clock rate is half the first-level comparison clock rate so that the first-level data comparison can be completed before starting the second-level comparison. Finally, the maximum value obtained after the second-level comparison is read out and stored in the storage module to complete the pooling. The input data is reduced by three layers of pooling, resulting in a final input size of 1 × 31.

### 3.3 EEG epilepsy detection circuit

**FIGURE 7:**
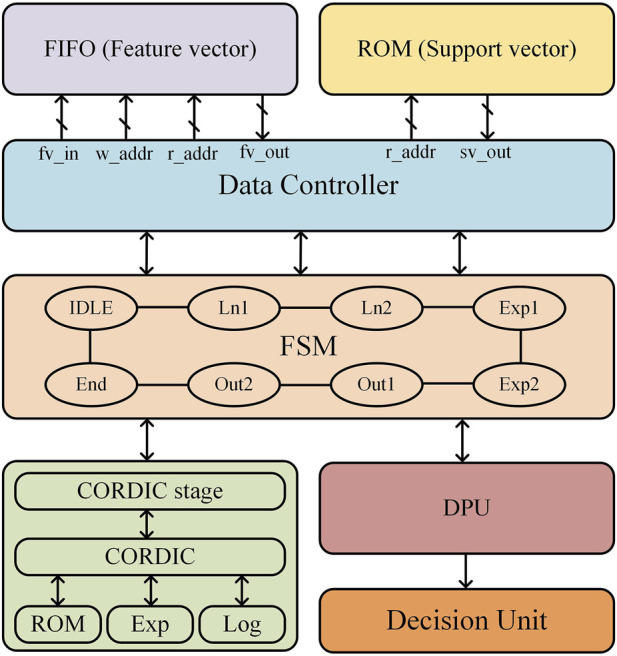
SVM hardware structure.

SVM is a common supervised learning algorithm mainly used for classification and regression problems; the basic idea is to find an optimal hyperplane in the feature space to separate different categories of sample points for better generalization performance (Cortes and Vapnik, 1995). In order to improve the accuracy of epilepsy classification, a SVM model based on Gaussian kernel function was developed. The SVM model was trained to obtain the decision function (Balestrini et al., 2021), which was then used to design the SVM hardware circuit structure, as shown in Figure 7. This structure consists of five main parts: the feature vector and support vector storage module, data read/write control module, finite state machine, coordinate rotation digital computer (CORDIC) module, and data processing unit (DPU). The input EEG data are extracted and stored in a FIFO buffer after the three-layer convolution module and the maximum pooling module as well as the pre-trained support vectors are stored in an off-chip ROM. At the beginning of classification, feature vectors and support vectors are sequentially read out through the data read/write control module, and logarithmic and exponential operations are completed in the CORDIC module under the control of a finite state machine, and then inputted into the DPU module and decision unit to derive the epilepsy classification results.

$$f\left(x\right)=\sum_{i=0}^{L-1}\alpha_{i}\exp\left(-\frac{{\left(\max s-\min s\right)}^{2}}{\sigma }\sum_{j=0}^{k-1}\frac{{\left(x_{j}-sv_{i,j}\right)}^{2}}{{\left(\max f-\min f\right)}^{2}}\right)-b$$ (1)

**FIGURE 8:**
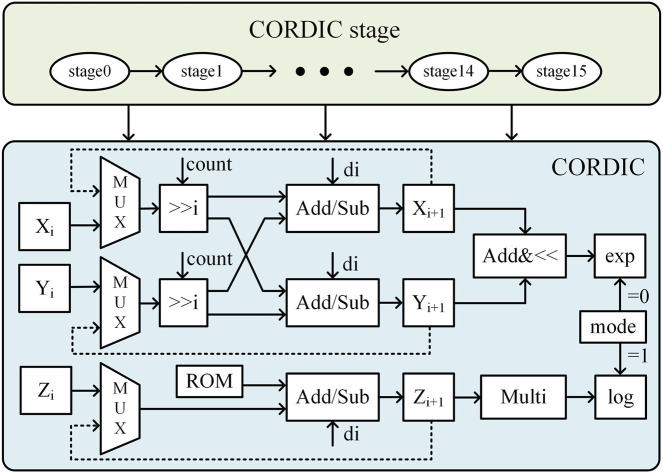
Hardware circuit diagram of the CORDIC algorithm.

The CORDIC algorithm is a highly efficient numerical computation method that is well-suited for hardware acceleration, as it replaces complex mathematical operations with a series of iterative addition, subtraction, and bit-shifting operations, significantly reducing computational complexity and hardware implementation costs. In this work, we design a single-precision floating-point exponential and logarithmic computation circuit based on the optimized CORDIC algorithm, improving both computational speed and accuracy while minimizing resource overhead. The hardware circuit of the CORDIC module is shown in Figure 8 and consists of three adders, two right shifters, and a ROM unit that stores the precomputed rotation angles for each iteration. Unlike conventional CORDIC implementations, our design integrates both rotational and vectoring modes to efficiently compute exponential and logarithmic functions, which are essential for SVM kernel function evaluation. This module performs two critical tasks: normalization of feature vectors and support vectors via logarithmic transformation and efficient computation of the exponential function in the classification decision function. The mode selection signal dynamically configures the circuit for logarithmic or exponential operations, optimizing hardware resource utilization. The pipelined iterative structure ensures high-speed execution, and after 16 iterations, the output undergoes post-processing under mode control to complete precise exponential and logarithmic calculations for SVM kernel function evaluation.

**FIGURE 9:**
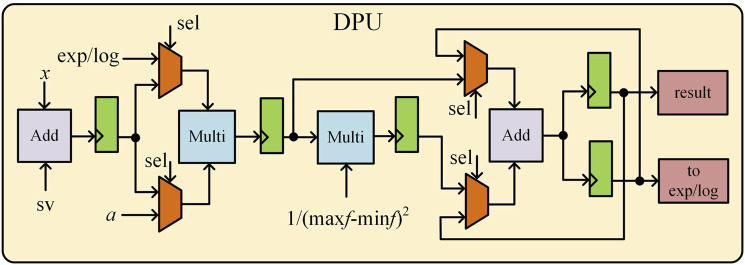
DPU hardware circuit diagram.

The DPU shown in Figure 9, is designed to efficiently compute SVM decision functions while minimizing computational overhead. The optimized hardware architecture consists of two adders, two multipliers, registers, and a binary selector, supporting both feature vector transformations and final classification decision computations. The mode selection signal (sel) enables adaptive switching between feature transformation mode and decision computation mode, ensuring optimal processing efficiency. In the mode of calculating the input of CORDIC module, after taking the logarithm, the feature vector is summed with the inverse code of the support vector, and then the result is input to the multiplier through the registers and selector to complete the squaring operation. The result is then normalized to obtain the input of the CORDIC module in exponential working mode. In the mode of calculating the result of the processing unit, firstly, the parameter *α*_i_ obtained from the model training and the output of the CORDIC module in exponential working mode are input into the multiplier at the same time to obtain the result of the multiplication, and after completing the multiplication and accumulation for a certain number of times, it is inputted into the decision-making unit, and is added to the inverse code of the parameter b to obtain the final result of the epilepsy classification.

## 4 Experimental results and analysis

In this study, experiments were conducted on an AMD Ryzen 5 3500U 2.10 GHz processor. The epilepsy detection model was trained in an environment configured with Anaconda3, TensorFlow 2.1, and Python 3.12 (CPU version). The hardware circuit design was implemented using the VCS simulation tool, Design Compiler for logic synthesis, and IC Compiler for physical layout.

### 4.1 Functional validation of epilepsy detection

**FIGURE 10:**
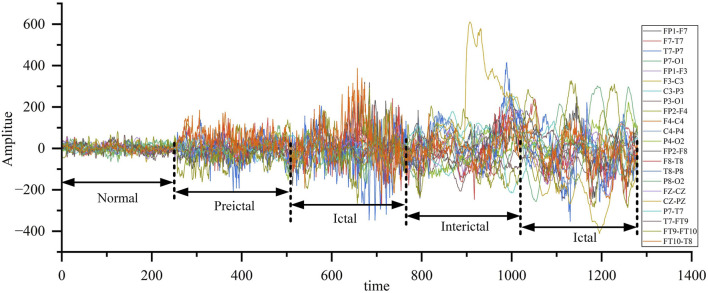
EEG waveform of patients with epilepsy at different stages.

Figure 10 shows the EEG waveforms of epileptic patients at different stages, including the normal stage, the preictal stage, the ictal stage, and the interictal stage. This study primarily focuses on the interictal and ictal stages, with epileptic seizure detection achieved by analyzing the differences in EEG waveform characteristics between these two stages.

Both the BONN EEG dataset and the CHB-MIT EEG dataset were used for training and testing. The BONN dataset was only used as a test set, while the CHB-MIT dataset was used for both training and testing. The CHB-MIT dataset consists of 23 recordings from 22 subjects, totaling 967.85 h of data with a sampling frequency of 256 Hz. During the data pre-processing stage, the EEG recordings, which are 1-h or 4-h long, were segmented into data samples of size 20 × 256 before training and testing. After segmentation, the CHB-MIT dataset contains 14,280 EEG samples, with 13,566 samples used for training and the remaining 714 samples, along with 500 samples from the BONN dataset, used for testing.

**FIGURE 11:**
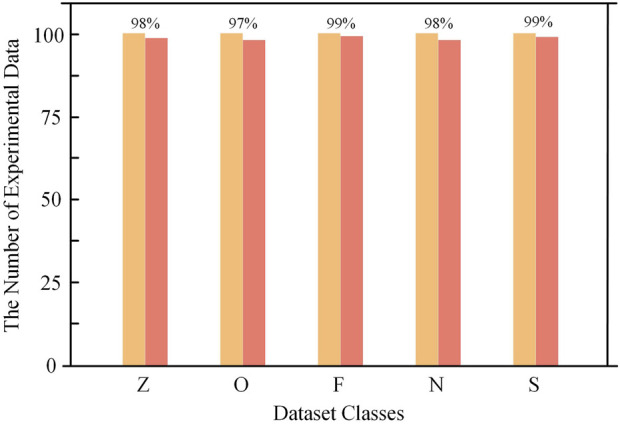
BONN dataset test results.

The seizure detection performance can be evaluated by the following three indicators, namely, sensitivity, specificity and accuracy, which were calculated as shown in Formulas 2–4.

$$Specificity=\frac{TN}{TN+FP}$$ (2)

$$Sensitivity=\frac{TP}{TP+FN}$$ (3)

$$Accuracy=\frac{TP+TN}{TP+TN+FP+FN}$$ (4)

where *TP* is true positive, *FP* is false positive, *TN* is true negative and FN is false negative. To validate the proposed CNN-SVM epilepsy detection model, this study conducted experiments and analyses using the BONN and CHB-MIT databases to assess the model’s capability to detect epilepsy. Experiments were conducted on five data subsets (*Z*, *O*, *F*, *N*, and *S*) from the BONN database, and the classification results are shown in Figure 11. Data subset *Z* and data subset *O*, which represent the EEG recordings from healthy volunteers with eyes open and eyes closed, achieved classification accuracies of 98% and 97%, respectively. Data subsets *F* and *N* correspond to interictal periods in epilepsy patients, while subset S represents ictal periods. The detection accuracies for these three subsets were 99%, 98%, and 99%, respectively.

**FIGURE 12:**
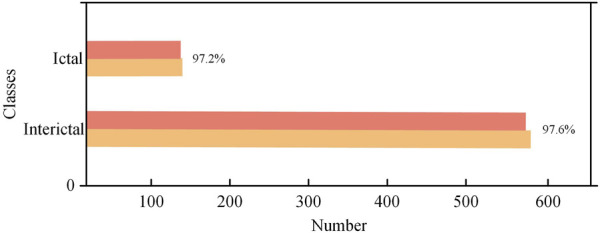
CHB-MIT dataset test results.

**FIGURE 13:**
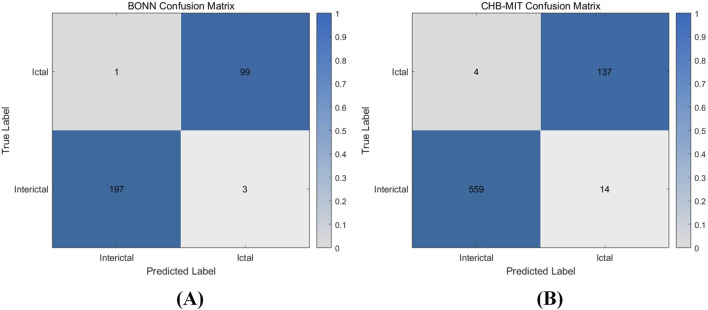
Confusion matrix of hardware classification results **(A)** BONN classification confusion matrix **(B)** CHB-MIT classification confusion matrix.

Figure 12 shows the test results of the CHB-MIT database, which contains 24 sets of data from 23 epilepsy patients (one of them participated in the recordings again 2 years later and contains two sets of data). Each recording contains multiple instances of interictal and ictal EEG signals. In the experiment, the data were preprocessed and categorized into two groups: interictal and ictal periods. The entire dataset was then input into the model for classification. The detailed classification results of the two datasets are shown in Table 3. For the 573 interictal instances, the model correctly detected 559 cases with 14 false positives, achieving a detection accuracy of 97.6%. For the 141 ictal instances, the model correctly identified 137 cases with 4 false positives, resulting in a detection accuracy of 97.2%. Figure 13 shows the confusion matrix for the model’s classification results for both datasets. These results, along with the confusion matrices, indicate that the model demonstrates strong classification performance across both datasets.

**TABLE 3:** Sample data test result.

Network Model	Dataset	Sample Size	Sample Type	Performance		
				Accuracy	Sensitivity	Specificity
CNN-SVM	BONN	300	Interictal Ictal	98.7%	99.0%	98.5%
CNN-SVM	CHB-MIT	714	Interictal Ictal	97.5%	97.2%	97.6%

### 4.2 Performance of epilepsy detection circuit

**FIGURE 14:**
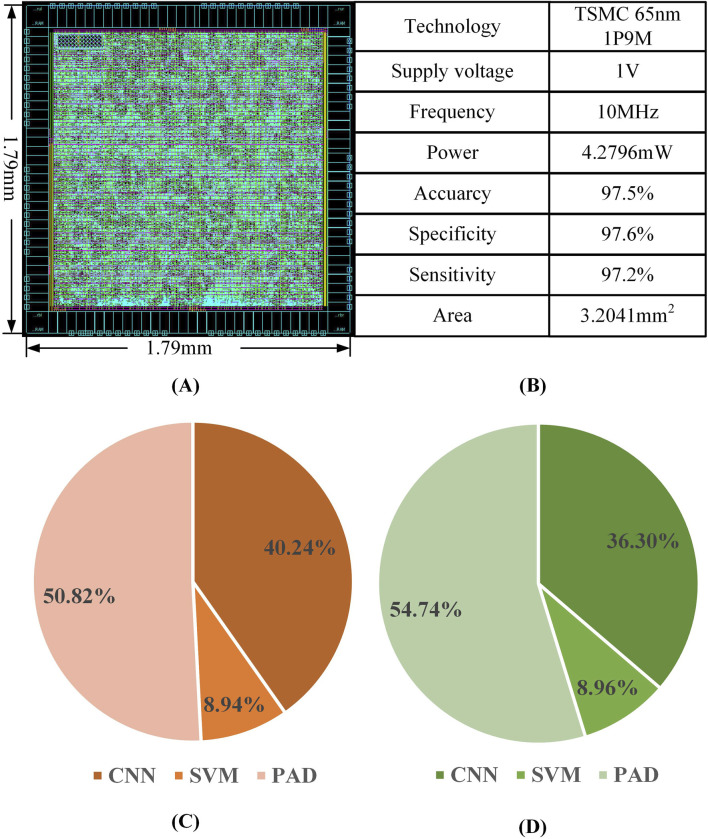
Epilepsy detection circuit **(A)** circuit layout **(B)** Key performance parameters **(C)** Area distribution of modules **(D)** Power distribution of each module.

The experimental results of the epilepsy detection circuit are illustrated in Figure 14. The back-end design of the epilepsy detection circuit was completed using the TSMC 65 nm process, resulting in an overall layout area of 1.79 × 1.79 mm^2^. Within this layout, the convolutional neural network module occupies 36.30% of the area, the support vector machine module occupies 8.96%, and the PAD occupies 54.74%. The overall power consumption of the circuit is 4.2796 mW at a power supply voltage of 1V and an operating frequency of 10 MHz, of which the power consumption of the convolutional neural network module accounts for 40.24%, that of the support vector machine module for 8.94%, and that of the PAD for 50.82%.

**FIGURE 15:**
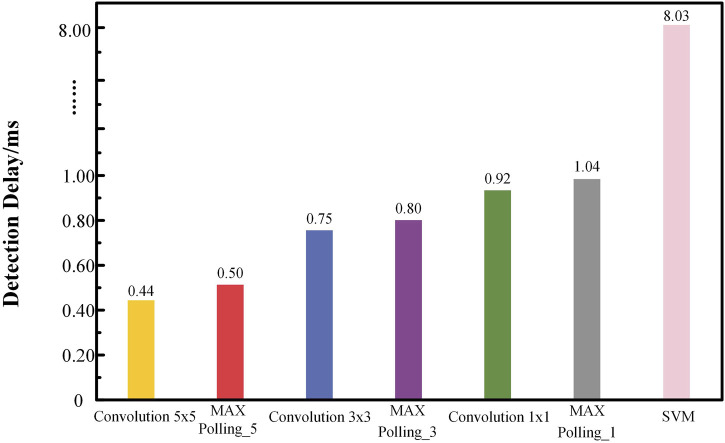
Cumulative delayed test results for epilepsy detection.

Figure 15 illustrates the cumulative processing time from input to output as the signal passes through the CNN-SVM hybrid AI model. The total detection delay for a single epilepsy detection instance in the hardware circuit is 8.03 ms (0.008 s). The processing begins with data input, followed by the first 5 × 5 convolutional layer, which takes 0.44 ms. This is followed by the first pooling layer, which takes 0.06 ms. The second convolutional layer, a 3 × 3 convolution, requires 0.25 ms, followed by the second pooling layer, which takes 0.05 ms. The third convolutional layer, a 1 × 1 convolution, takes 0.12 ms, and the third max pooling and data output together require 0.12 ms. Finally, the extracted features are fed into the SVM classifier, which has a processing delay of 6.99 ms. As a result, the total detection time amounts to 8.03 ms.

### 4.3 Comparison with other works

Table 4 summarizes the performance comparison between the proposed CNN-SVM hybrid model and other similar works. An epilepsy detection system based on the Hjorth descriptor method for feature extraction and KNN classification was implemented on a Zynq7 series FPGA, achieving a classification accuracy of 90.74% with a classification delay of 0.015 s (Rizal et al., 2022). A nonlinear support vector machine classification system was developed using a Virtex5 series FPGA, incorporating a discrete wavelet transform feature extraction module, an improved sequence minimum optimization training module, and a nonlinear SVM module, achieving an accuracy of 94.2% (Wang et al., 2018). A RISC-V CNN coprocessor for epilepsy detection was designed with low-power techniques to reduce system power consumption while implementing epilepsy detection through an 11-layer CNN network (Lee et al., 2017). A Seizure-Cluster-Inception CNN (Sci-CNN) was proposed to address the epilepsy classification specificity problem, and the SRAM access rate was reduced through Kernel-Wise Pipeline (KWP), achieving a classification accuracy of 93.0% but with a maximum latency of 17 s (Tsai et al., 2023). A sequence minimization optimization algorithm was used to train an SVM-based epilepsy classifier, achieving 96.7% classification sensitivity, 90.43% specificity, and a system power consumption of 14.91 mW on a 65 nm ASIC (Elhosary et al., 2019). In comparison, the hybrid AI network hardware circuit model proposed in this work achieves a classification accuracy of 97.5% and a sensitivity of 97.2% at 65 nm, demonstrating good generalization capability while achieving a single epilepsy detection delay of only 0.008 s.

**TABLE 4:** Comparison with other works.

	Rizal et al. (2022)	Wang et al. (2018)	Lee et al. (2017)	Tsai et al. (2023)	Elhosary et al. (2019)	This Work
Technology/FPGA	Zynq7	Virtex5	180 nm	40 nm	65 nm	65 nm
Area	N/A	N/A	2.18 mm^2^	0.114 mm^2^	0.20 mm^2^	3.204 mm^2^
Voltage	N/A	N/A	1.8 V	1.8 V	1.2 V	1 V
Accuracy	90.74%	94.2%	97.8%	93.0%	90.3%	97.5%
Sensitivity	N/A	92.2%	90.4%	90.4%	96.7%	97.2%
Latency	0.015 s	N/A	0.012 s	17 s	N/A	0.008 s
Power	N/A	N/A	0.1 mW	N/A	14.91 mW	4.27 mW

Compared with related works, the proposed CNN-SVM hybrid hardware circuit demonstrates higher computational efficiency and lower latency. Specifically, our design achieves a classification latency of 0.008 s, which is lower than 0.015 s in (Rizal et al., 2022) and 0.012 s in (Lee et al., 2017), representing a 46.7% improvement over (Rizal et al., 2022) and a 33.3% improvement over (Lee et al., 2017). Compared with (Tsai et al., 2023), the latency advantage is even more significant, as (Tsai et al., 2023) reports a maximum delay of 17 s, while our design completes epilepsy detection in just 0.008 s. This reduction in processing time is attributed to the hardware-optimized CNN-SVM architecture, which enhances data flow efficiency and minimizes computational overhead.

Furthermore, our model maintains a classification accuracy of 97.5% and sensitivity of 97.2%, showing better generalization capability compared to several previous FPGA and ASIC implementations. Compared with (Elhosary et al., 2019), which also adopts a 65 nm process and an SVM-based classifier, our approach achieves higher accuracy (97.5% vs 90.3%) while ensuring significantly lower latency.

In terms of power efficiency, our design consumes 4.27 mW, which is significantly lower than the 14.91 mW reported in (Elhosary et al., 2019). Although (Lee et al., 2017) achieves lower power consumption (0.1 mW), it is implemented on 180 nm technology, whereas our design is based on 65 nm, balancing high accuracy (97.5%) and power efficiency.

Generally speaking, the proposed CNN-SVM hybrid AI model demonstrates several advantages in practical applications. By utilizing a pipelined convolutional computation circuit and a parallel-style row computation method, the model achieves a low-latency detection of 0.008 s, which is crucial for real-time epilepsy monitoring. Compared to non-pipelined designs, the pipelined convolution architecture significantly enhances computational efficiency by allowing different stages of convolution—multiplication, accumulation, and activation—to operate concurrently. In a non-pipelined system, each convolution operation must be completed before the next one begins, resulting in idle computation cycles and increased latency. In contrast, the proposed pipelined approach ensures continuous data flow, where multiple convolution operations are processed in overlapping phases, effectively reducing the overall processing time while maintaining high accuracy.

Additionally, the optimized max pooling strategy plays a crucial role in improving classification accuracy. Unlike average pooling, which smooths out feature variations, max pooling retains the most significant activations, preserving critical EEG features that contribute to epilepsy classification. However, traditional max pooling designs introduce memory access bottlenecks, increasing computational delays. To mitigate this, our implementation employs a parallelized comparison mechanism that efficiently selects the maximum value within each pooling window in a single step. Furthermore, a register-based caching strategy minimizes redundant memory accesses, reducing power consumption while maintaining high-speed processing. These enhancements ensure that the feature extraction process remains efficient and robust against EEG signal variations.

To further enhance computational efficiency, we implement a 32-bit single-precision floating-point CORDIC computation unit for exponential and logarithmic calculations in the SVM classification module. Traditional methods rely on complex floating-point multiplications and divisions, which introduce significant computational overhead and power consumption. The CORDIC algorithm replaces these costly operations with a series of iterative shift-and-add calculations, significantly reducing hardware complexity. By using a three-adder, two-shifter architecture with a ROM-based angle lookup table, our design efficiently computes exponential and logarithmic functions required for the Gaussian kernel SVM, eliminating the need for resource-intensive floating-point units. This optimization enables real-time execution of the SVM decision function, ensuring fast and energy-efficient classification.

## 5 Discussion

In this study, we propose a high-accuracy and low-cost epilepsy detection hard-ware circuit utilizing a hybrid AI network model embedded in a SoC for EEG signal analysis. Memory storage requirements are optimized through IEEE754 single-precision floating-point encoding, while data remapping techniques alleviate memory access pressure. To enhance computational efficiency, hierarchical processing is implemented for multiply-accumulate operations in the hybrid network. A configurable convolution layer with parallel row computation and pipelined algorithms accelerates feature extraction, achieving a 32% reduction in latency compared to existing literature. Additionally, a CORDIC-based computation circuit is designed to expedite exponential and logarithmic operations, improving both speed and accuracy. Experimental results demonstrate a compact chip area of 3.20 mm^2^ under TSMC 65 nm technology, power consumption of 4.28 mW at 10 MHz frequency, and an ultra-low detection latency of 0.008 s. The model achieves 97.5% accuracy, 97.6% sensitivity, and 97.2% specificity on the CHB-MIT dataset, outperforming traditional methods and hardware implementations. Compared to MRI-based approaches, which rely on spatial-domain brain structure imaging with high computational overhead, our EEG-centric design captures temporal dynamics of neural activity at significantly lower hardware resource costs. While MRI excels in spatial correlation analysis, EEG enables real-time monitoring of electrophysiological changes. Multimodal fusion of these complementary techniques could further enhance diagnostic comprehensiveness.

Against state-of-the-art solutions, our work reduces power consumption by 23.8% and area by 18.6% through innovations such as shared PE units for CNN-SVM operations and kernel-wise pipeline optimization. The configurable convolution layer supports 5 × 5, 3 × 3, and 1 × 1 kernels with hardware resource reuse, while the two-stage max pooling circuit minimizes data dimensionality without compromising feature integrity. These advancements position the proposed circuit as a viable solution for portable, real-time epilepsy detection devices.

### 5.1 Limitations

Despite the proposed hybrid artificial intelligence network model achieving an accuracy of 97.5%, limitations regarding overfitting and generalization across diverse populations still need to be considered. The study’s reliance on specific datasets (Bonn and CHB-MIT) may limit the applicability of the research findings to a broader population. Future work should consider incorporating more datasets and conducting more extensive validation to enhance the model’s robustness and ensure its effectiveness across different populations.

### 5.2 Future work

In future research, we could further explore different AI architectures or integrate other physiological signals to improve the accuracy of epilepsy detection. For instance, they might consider using other neural network structures in deep learning, such as Recurrent Neural Networks (RNNs) or Transformers, which have unique advantages in processing time - series data and could potentially enhance the analysis of EEG signals. Moreover, integrating other physiological signals, such as heart rate variability (HRV) or electromyography (EMG), might provide additional information for epilepsy detection, thereby improving the accuracy and reliability of the detection. Through these approaches, it is expected that the epilepsy detection model could be further optimized, offering stronger support for clinical diagnosis and treatment.

In addition, our chip design at the 65 nm process can be ported to more advanced processes. Subsequently, by integrating a low-power communication module, it can transmit information in real-time to the nearest hospital when an epileptic patient has a seizure, enabling timely medical intervention and treatment.

## 6 Conclusion

A hybrid AI network hardware circuit with high accuracy and low cost-latency is proposed for the detection and classification of epilepsy in portable medical devices. The model consists of three convolutional layers, three pooling layers and one support vector machine module. Epilepsy detection is realized by SVM classification. The hardware data adopts the single-precision floating-point number standard, and a configurable convolution layer for parallel computing is designed to optimize the reuse of hardware resources. In addition, a pipeline convolution algorithm is used to accelerate the convolution operation. A CORDIC algorithm based on 32-bit floating-point operations is designed to speed up exponential and logarithmic operations and improve data accuracy. The hardware design is based on the TSMC 65 nm process, with an operating voltage of 1 V, an operating frequency of 10 MHz, and a power consumption of 4.2796 mW. The BONN and CHB-MIT datasets were used for classification testing, with the classification accuracies of the model being 98.7% and 97.5%, respectively, providing good classification accuracies.

## Data Availability

The original contributions presented in the study are included in the article/supplementary material, further inquiries can be directed to the corresponding authors.
